# Infusion of blood from young and old mice modulates amyloid pathology

**DOI:** 10.18632/aging.206319

**Published:** 2025-09-12

**Authors:** Matias Pizarro, Ruben Gomez-Gutierrez, Ariel Caviedes, Catalina Valdes, Ute Woehlbier, Cristian Vargas, Mauricio Hernandez, Claudia Duran-Aniotz, Rodrigo Morales

**Affiliations:** 1 Latin American Institute for Brain Health (BrainLat), Universidad Adolfo Ibáñez, Santiago, Chile; 2 Center for Social and Cognitive Neuroscience (CSCN), School of Psychology, Universidad Adolfo Ibanez, Santiago, Chile; 3 Department of Neurology, The University of Texas Health Science Center at Houston, Houston, TX 77030, USA; 4 Center for Integrative Biology (CIB), Universidad Mayor, Santiago, Chile; 5 Division of Biotechnology, MELISA Institute, San Pedro de la Paz, Bio-Bio, Chile; 6 Centro Integrativo de Biología y Química Aplicada (CIBQA), Universidad Bernardo O’Higgins, Santiago, Chile

**Keywords:** Alzheimer’s disease, amyloid-β, neurodegeneration, protein misfolding, blood infusion, therapeutic targets

## Abstract

Alzheimer’s disease (AD) is a neurodegenerative disease characterized by the accumulation of misfolded proteins in the brain. Recently, the impact of blood components in the progression of this disease has come to attention. This study investigates the effects of infusing blood from young and old wild-type mice into transgenic mice that model AD brain amyloidosis. Impaired memory and Aβ accumulation were observed in mice infused with blood from old donors. A proteomic analysis in the brain of these mice identified alterations in components related to synaptogenesis and the endocannabinoid system. The α2δ2 protein, associated with neuronal calcium regulation, was validated as a possible mediator of the observed effects. This study highlights the influence of blood in AD pathology and the identification of potential therapeutic targets.

## INTRODUCTION

Alzheimer’s disease (AD) is a progressive and devastating neurodegenerative disease that affects older individuals. AD is pathologically characterized by the accumulation of misfolded amyloid-β (Aβ) and tau proteins in the brain [1]. Notably, misfolded Aβ peptides are considered important pathological mediators in AD due to their intrinsic toxicity and profuse extracellular deposition in senile, diffuse, and vascular plaques [2]. From a pathological standpoint, Aβ aggregates are considered the earliest abnormality in AD, triggering subsequent changes leading to neurodegeneration and disease onset [3, 4]. Experimental evidence and data collected from human subjects strongly suggest that the accumulation of misfolded Aβ triggers tau pathology, synaptic dysfunction, neuronal death, and cognitive decline [5, 6]. Among the diverse array of misfolded Aβ species in the AD brain, oligomers are linked with initial seeding stages [7, 8] and enhanced toxic activities [9].

For many years, Aβ aggregates were believed to be confined in the brain [10]. However, some studies strongly suggest their presence in peripheral tissues, such as the retina, heart, skin, blood vessels, and gastrointestinal tract [11–14]. These peripheral aggregates have gained increasing attention in AD research as they may contribute to the spread and dissemination of amyloid pathology [12, 15]. The repercussions of peripheral Aβ accumulation are potentially multifaceted as evidence indicates that they can contribute to the development of AD through several mechanisms [16]. Therefore, the presence of Aβ in peripheral tissues and circulation may contribute to disrupt peripheral clearance mechanisms, leading to increased Aβ levels in the brain and contributing to the development of brain amyloidosis [17]. Along this line, the concept of blood based Aβ clearance has emerged as a therapeutic strategy [18]. Some studies have found that Aβ clearance in the periphery can substantially reduce Aβ accumulation in the brain [16, 19, 20]. Notably, the implementation of plasma albumin exchange has demonstrated a marked reduction in Aβ burden in AD patients, accompanied by improvements in AD-related cognitive function [21–23]. Furthermore, alternative techniques such as hemodialysis and peritoneal dialysis have also shown promise in decreasing Aβ levels in brain [16, 19, 24]. These findings underscore the diverse approaches that leverage blood-based mechanisms for Aβ clearance, providing valuable insights for the development of effective therapeutic interventions [25, 26].

In the context of blood-based strategies to treat AD, several studies have investigated the potential benefits or detrimental effects of young and old blood donors on aging. These studies have described significant effects in blood recipients, suggesting that the factors carried by young and old blood can modulate biological functions in beneficial or detrimental manners [27–30]. Initial studies involving heterochronic parabiosis reported notable beneficial changes at systemic levels in old animals, strongly suggesting the presence of regenerative factors in young blood [31]. Considering this and other data, young blood became the focal point of numerous animal and human studies, delving into its potential benefits for brain health and aging [31–33]. Recent studies have shown that the blood from young mice provides therapeutic benefits relevant to aging and brain diseases. For example, infusions of young blood can reverse the effects of brain aging at the synaptic level, increase dendritic spine density and plasticity in the hippocampus, and improve age-related cognitive impairments [34]. Also, plasma from young wild type mice reduces phosphorylated tau and tau tangles in the brain [35]. Likewise, the introduction of young blood to aged animals, either through parabiosis or young plasma infusion, induces a restoration in the levels of synaptic and neuronal proteins, consequently improving memory in aged mice [36, 37]. Moreover, a reduction in tau and Aβ pathologies, coupled with diminished brain inflammation, has been observed as a consequence of similar treatments [38]. Another study using whole blood exchange from young wild type mice into transgenic mice from 3 to 13 months-old shows a reduction in Aβ burden and memory improvement [39]. Interestingly, the authors found a persistent effect as recorded up to 17 months of treatment [40].

Despite the above-mentioned evidence, the impact of old blood transfusion on AD pathology remains understudied. One study described that plasma and platelets from aged APP/PS1 mice increased brain Aβ deposition and learning/memory deficits when infused into younger animals. In addition, the introduction of aged platelets elevated Aβ1-40, Aβ1-42, and tau protein levels in the brain of treated mice [41]. Another study, using heterochronic blood exchange, showed that aged mouse blood induces aging phenotype in younger mice; however, brain senescence parameters were not altered [31, 42]. Particularly, our group infused young Tg2576 transgenic mice (transgenic mice expressing human amyloid precursor protein (APP)) with whole blood or plasma from older Tg2576 mice, which resulted in increased brain amyloidosis and neuroinflammation in the recipient mice [41]. Likewise, the intravenous administration of purified Aβ aggregates sped up amyloid pathology and triggered neuropathological changes, supporting the idea that bloodborne Aβ seeds are capable of triggering neuropathological changes [13, 43]. Interestingly, one of these studies [41] did not show increases in amyloid pathology when young mice were infused with blood from old wild type mice. However, it is relevant to note that these mice received limited (either one or two) doses of low-volume blood infusions.

In summary, most published studies in this area have focused on the potential therapeutic effects of young blood infusion to treat AD. However, few studies have examined the potential detrimental effects of old blood on brain amyloidosis, and even fewer have explored the impact of blood from wild type mice. Additionally, the potential role of blood transfusion in modulating Aβ accumulation, inflammation, and behavior, and its subsequent impact on AD pathology, is an intriguing avenue to explore. This study aimed to fill these gaps by examining the effects of a long-term blood infusion regime from young and old wild type donors into mice that model brain amyloidosis. We also evaluated the effect of these treatments in other associated detrimental events including neuroinflammation and cognitive decline.

## RESULTS

### Behavioral differences in Tg2576 mice infused with blood from young or old wild-type donors

**Figure 1:**
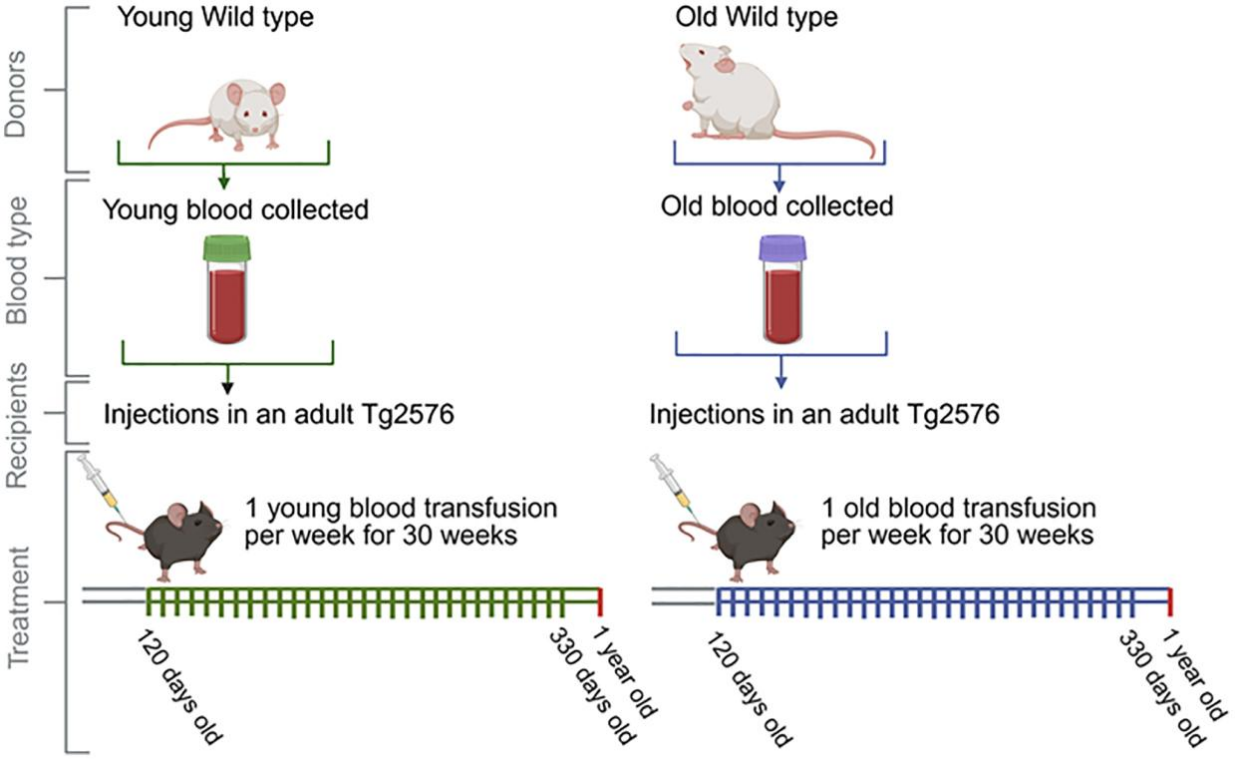
**Schematic representation of the blood infusion regime (blood from old and young wild type mice into Tg2576 mice).** Wild-type mice aged 50–75 days (WT Young mice), and wild-type mice aged 443–532 days (WT Old mice) served as blood donors. This blood was transfused to 120-day-old Tg2576 mice, which then underwent to weekly transfusions and sacrificed at 363–366 days old. Before sacrificing, mice were evaluated for spatial memory. *Postmortem* analyses included immunopathological, biochemical, and proteomic evaluations of brain tissues.

**Figure 2:**
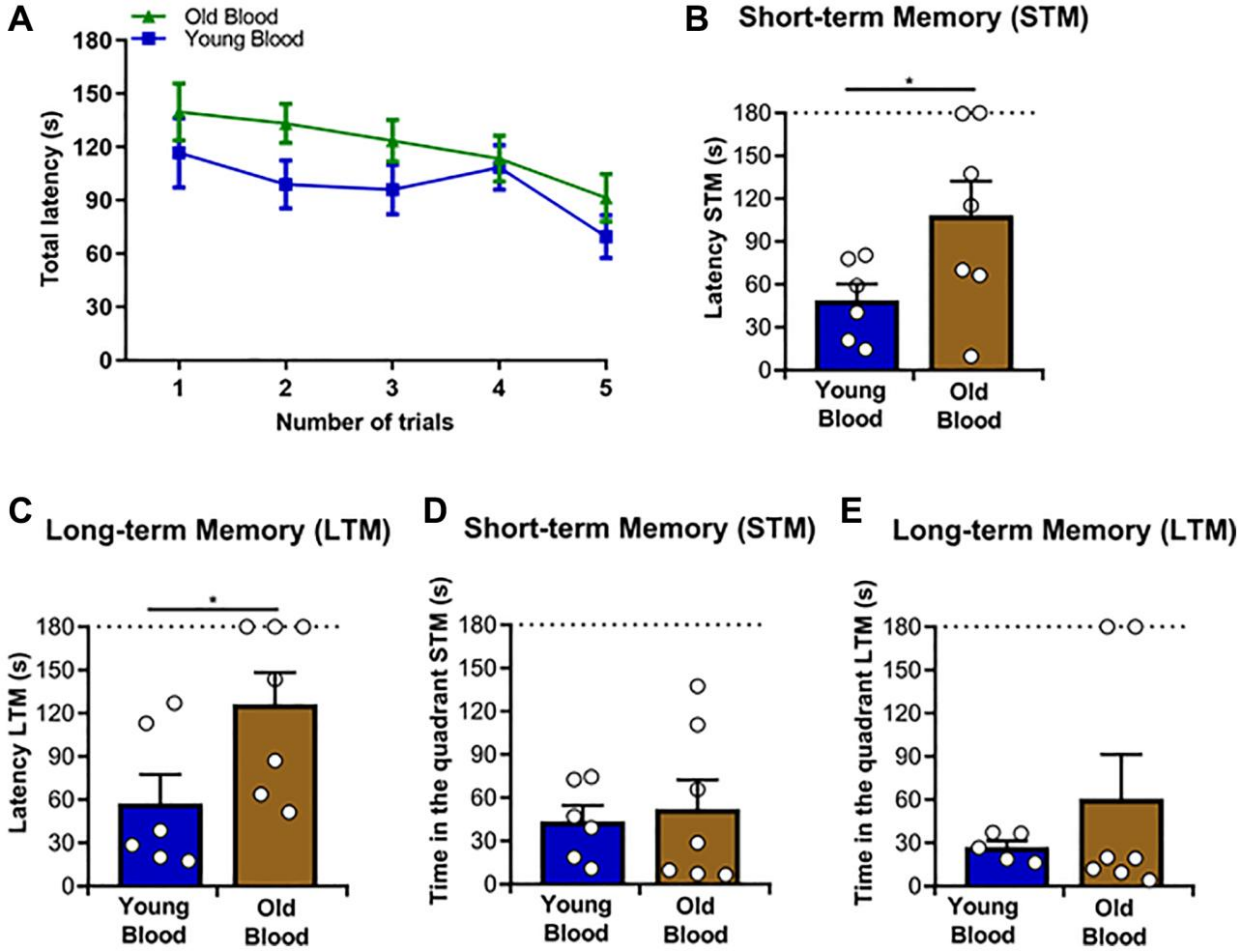
**Spatial memory analyses of Tg2576 mice treated with blood from old and young wild type mice.** The Barnes maze test was applied to all mice included in this study. (**A**) Average latency to the escape chamber for the training trials sessions over 5 days, as described in Materials and Methods. (**B**) Quantitative analysis of the short-term memory (STM) latency. (**C**) Quantitative analysis of the long-term memory (LTM) latency. (**D**) Quantitative analysis of the STM time-in-quadrant parameter. (**E**) Quantitative analysis of the LTM time-in-quadrant parameters. *N* = 6–7/group, (random mix of males and females; young donor group: 3M/3F; old donor group: 3M/4F). Sex was not included as a variable in statistical analyses. Data values are expressed as means ± SEM. Data in (**A**) were analyzed using repeated/measures ANOVA. Data in (**B**) and (**D**) were analyzed using Student’s *t*-test. Data in (**C**) and (**E**) were analyzed using the Mann-Whitney *U*-test. ^*^p < 0.05.

Multiple reports described that the administration of old blood components can transfer aging associated traits to younger individuals [28, 42, 44, 45]. On the contrary, the infusion of young blood is reported to provide beneficial effects over multiple deleterious phenotypes associated with aging [46, 47]. Considering that aging is the main risk factor of AD [48, 49], the effect of aged or young blood has been separately investigated in the context of this neurological disorder [50, 51]. Here, we aimed to study, in parallel, the effects that infusion of blood from old or young donors exerted in an animal model of brain amyloidosis. Specifically, we used Tg2576 mice [52] for these experiments. Male and female Tg2576 mice received blood infusions from either old or young wild-type animals from the same genetic background as described in the Materials and Methods section. Briefly, Tg2576 mice received 30 blood infusions at weekly intervals and sacrificed at 12 months of age. Then, their brains were studied for multiple disease-associated parameters including histopathological, biochemical, and proteomic evaluations (Figure 1). Before sacrificing, treated mice were tested for spatial memory using the Barnes maze paradigm [38]. Mice subjected to this test showed similar learning curves without significant differences between the groups treated with young or old blood (Figure 2A). Then, parameters for short-term memory (STM) and long-term memory (LTM) were measured. These analyses showed significant differences in latency for STM and LTM (Figure 2B, 2C). These results indicate that mice treated with old blood display more difficulties remembering where the escape chamber was located compared to subjects treated with blood from young wild type mice. The time mice spent in the target quadrant was also measured. There, no significant differences were registered when the test was conducted at 5 (STM) or 12 (LTM) days after starting training (Figure 2D, 2E). Overall, this information shows that blood from old or young donors modulate spatial memory when administered into a mouse model of AD.

### Infusion of blood from old or young wild type donors modulates amyloid pathology deposition in Tg2576 mice

**Figure 3:**
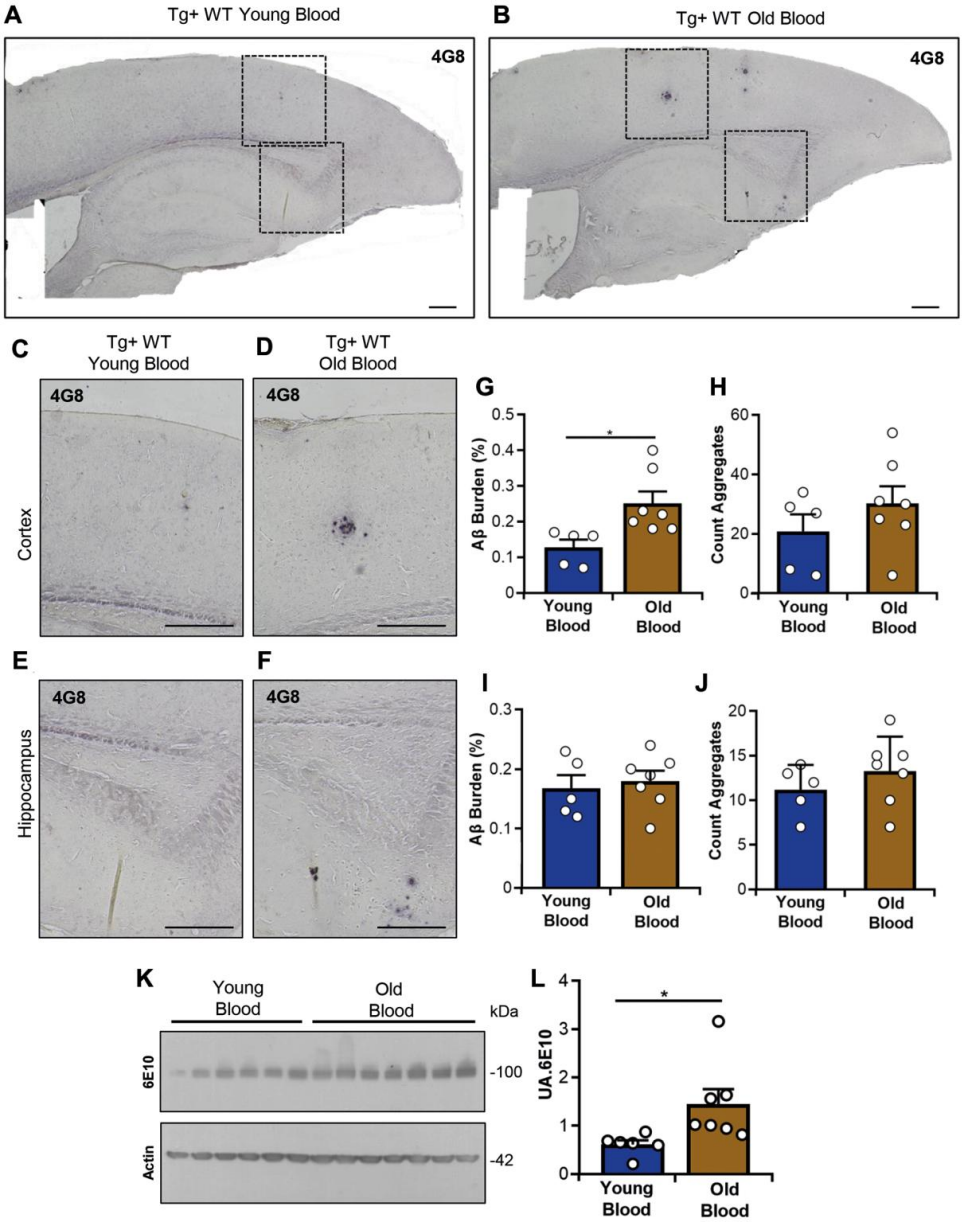
**Evaluation of Aβ deposition and APP levels in Tg2576 mice treated with blood from old and young wild type mice.** Representative images of Aβ accumulation in the cortex and hippocampus (**A**, **B**). Higher magnification images of the cerebral cortex (**C**, **D**) and hippocampus (**E**, **F**) are also shown. Tissue slices were probed with the 4G8 antibody as described in the Materials and Methods. Scale bars: 1,000 μm (**A**, **B**), 500 μm (**C**–**F**). Quantitative analyses of Aβ burden and plaque number in the cerebral cortex (**G**, **H**) and hippocampus (**I**, **J**) are displayed. (**K**) Representative western blot image showing APP levels in brain homogenates from blood-treated mice (upper panel), with actin used as a loading control (lower panel). (**L**) Densitometric quantification of APP levels shown in (**K**), expressed in arbitrary units (UA). Data include 5–7 animals per group, (random mix of males and females; young donor group: 1–3M/2–3F; old donor group: 1–3M/2–4F). Sex was not included as a variable in statistical analyses. Data values are expressed as mean ± SEM. Molecular weight markers (KDa) are indicated. Each lane represents one individual animal. Statistical analyses: Mann–Whitney *U*-test for panels (**G**) and (**L**); Student’s t-test for panels (**H**), (**I**), and (**J**). ^*^p < 0.05, ^**^p < 0.01.

Histological staining using the anti-Aβ 4G8 antibody and thioflavin S (ThS) was employed to observe amyloid deposition in hippocampal and cortical brain areas (Figure 3 and Supplementary Figure 1). These brain regions were specifically studied as they are the most affected in this mouse model and show increased amyloid pathology over time (Supplementary Figure 2). The extent of amyloid deposition using both analyses was compared and quantified between mice injected with young blood and those injected with blood from old mice. The analysis was conducted in terms of the area reactive to Aβ deposits in each brain region per the total analyzed area. The number of deposits in cortex and hippocampus was also measured. When examining amyloid depositions through 4G8 staining, a significant increase of Aβ deposits was observed in the brain cortices of Tg2576 mice treated with blood from old wild-type mice compared to brain cortices of Tg2576 mice treated with young wild-type blood (Figure 3A–3F). Interestingly, amyloid pathology in the hippocampi of the same mice did not show significant changes (Figure 3A, 3B, 3G–3J). We used similar analyses for the ThS-stained brain slices (Supplementary Figure 1). There, no significant differences between the groups were observed, suggesting that the differences observed in the mice’s brain cortices are mostly associated with diffuse Aβ deposition. To further examine the impact of blood infusion on brain amyloidosis, we quantified the total levels of Aβ_40_ and Aβ_42_ levels in brain homogenates from the same Tg2576 mice treated with blood from young and old wild-type mice. This analysis showed no significant differences between the groups when measuring Aβ_40_, Aβ_42_, or the Aβ_42_/Aβ_40_ ratios (Supplementary Figure 3). The discrepancy between this analysis and that conducted by IHC further suggests differences in the compactness of the Aβ plaques in these animals. Studies to analyze this possibility (e.g., denaturation profiles using different concentrations of denaturing agents, sucrose gradient fractionations, and others) will be conducted as part of future studies.

We additionally analyzed whether blood infusion altered other elements of the amyloid cascade. Specifically, we questioned whether the production of the amyloid-β precursor protein (APP) is affected by blood treatments. APP levels in the brain of mice treated with blood from both young and old wild-type donors were evaluated via western blotting using the 6E10 antibody. Interestingly, APP levels differed significantly between both treatment groups, with a notable increase in Tg2576 mice receiving old blood compared to mice who received young blood (Figure 3K, 3L). Overall, our data suggest that the infusion of blood from donors of different ages alters the deposition of Aβ in the brain of Tg2576 mice in a region-specific manner. These changes seem to be translated in the degree of compactness of the aggregates and could be related with the differential expression of APP in this particular transgenic model.

### Effect of blood infusion from wild type to Tg2576 mice in markers of glial cells

AD pathology is classically associated with brain inflammation. This is easily identified by an increase in activated astrocytes and reactive microglia [53]. We analyzed the degree of glial activation in the brain of mice treated with the different blood sources used in this experiment. This was achieved by immunohistochemical analyses targeting the glial fibrillary acidic protein (GFAP, an astrocytes marker, Supplementary Figure 4). A quantitative analysis of the GFAP staining was performed to assess the density of signal per unit area. No significant differences were observed, suggesting that the blood infusion treatments conducted in this study did not have an effect in the activation of brain astroglial cells.

### Infusion of blood from old or young wild type mice induce dysregulation in proteins involved in synaptic signaling pathways in Tg2576 subjects

**Figure 4:**
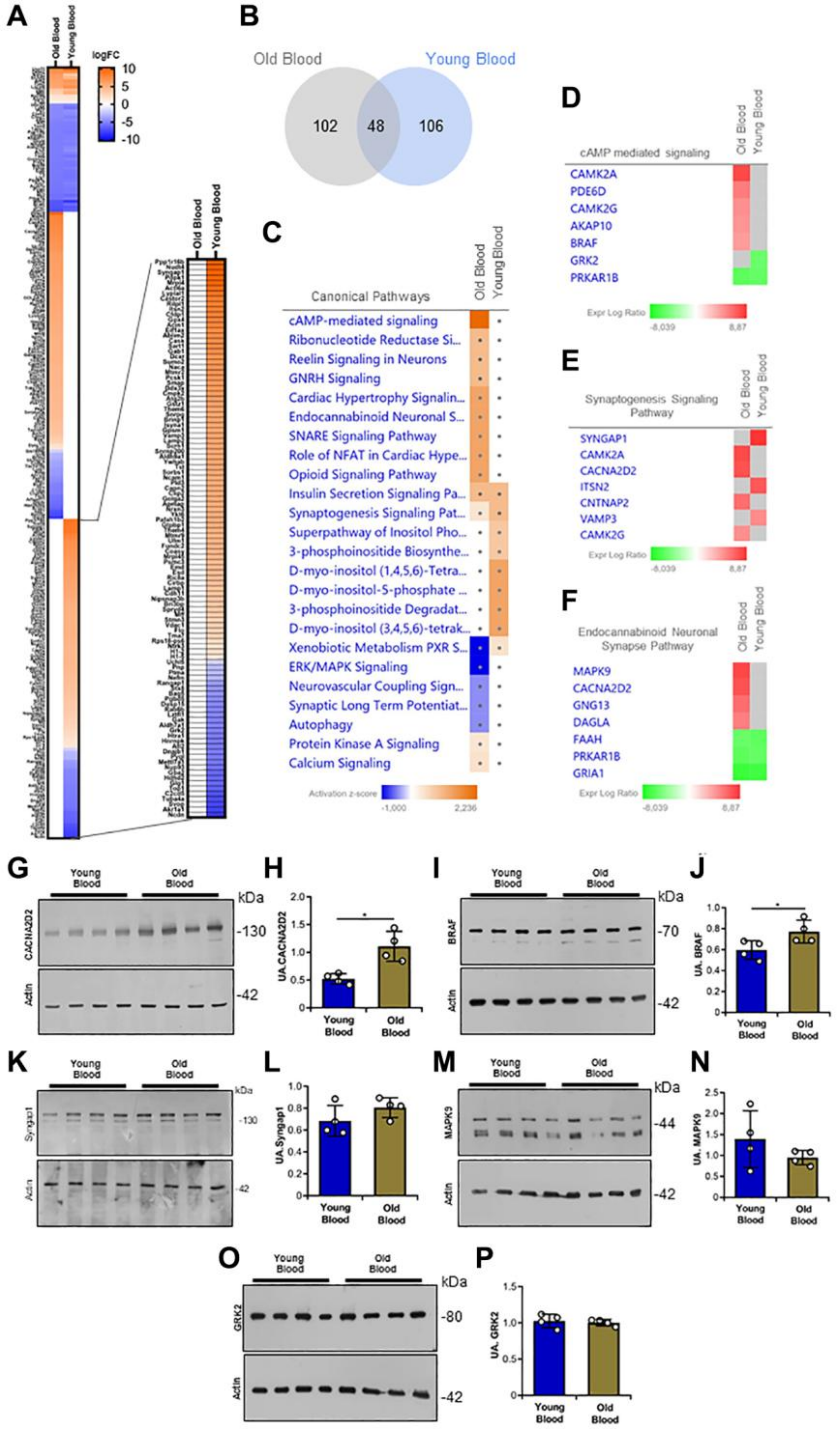
**Proteomic analysis of Tg2576 mice infused with blood from old and young wild type mice.** (**A**) Heatmap of quantifiable proteins from the Old Blood and Young Blood groups, showing log fold-change (logFC) in protein expression. (**B**) Venn diagram of differentially expressed proteins in an Old Blood vs. Young Blood groups comparison. (**C**) Canonical pathway analysis of differentially expressed proteins, highlighting enriched signaling pathways with activation z-scores. (**D**–**F**) Differentially expressed proteins in cAMP-mediated signaling, synaptogenesis signaling, and endocannabinoid neuronal synapse pathways, respectively. Gene names are used. (**G**, **H**) Representative western blot image and quantitative analysis of expression of CACNA2D2 in brain homogenates. (**I**, **J**) Representative western blot image and quantitative analysis of expression of BRAF in brain homogenates. (**K**, **L**) Representative western blot image and quantitative analysis of expression of Syngap1 in brain homogenates. (**M**, **N**) Representative western blot image and quantitative analysis of expression of MAPK9 in brain homogenates. (**O**, **P**) Representative western blot image and quantitative analysis of expression of GRK2 in brain homogenates. *N* = 3/group for proteomic analysis, and *n* = 4/group for protein validation (random mix of males and females; young donor group: 1–2M/2F; old donor group: 1–2M/2F). Data are expressed as mean ± SEM. Data in (**H**), (**J**), (**L**), (**N**), and (**P**) were analyzed using Student’s *t*-test. ^*^p < 0.05.

To further investigate the possible causes leading to spatial memory differences between Tg2576 mice infused with old and young blood, proteomic analyses were performed. For this purpose, brain homogenates from Tg2576 animals subjected to the blood transfusion regimen shown in Figure 1 were subjected to mass spectrometry. Once the total proteins present in the homogenate were identified, an analysis of the identified quantifiable proteins was performed. This analysis provided a total of 3,312 proteins (Figure 4A and Supplementary Data 1). Based on the above results, an analysis of Differentially Expressed Proteins (DEPs) was performed in Tg2576 mice infused with blood from either Old Wild Type and Young wild Type donors. This yielded a total of 256 DEPs (Figure 4B). IPA pathway analysis identified that several of the differentially expressed proteins were predicted to affect the cAMP mediated signaling (CAMK2A, PDE6D, CAMK2G, AKAP10, BRAF, GRK2, PRKAR1B), the synaptogenesis signaling pathways (SYNGAP1, CAMK2A, CACNA2D2, ITSN2, CNTNAP2, VAMP3, CAMK2G), and the neuronal endocannabinoid synaptic pathway (MAPK9, CACNA2D2, GNG13, DAGLA, FAAH, PRKARB1B, GRIA1) (Figure 4C–4F and Supplementary Figure 5). Based on the differentially expressed proteins in each pathway, and considering published evidence [54], candidate proteins for validation were selected. One of these proteins included the SYNGAP1 protein. This is a Ras GTPase activator protein, which exerts negative regulation on Ras, Rap and alpha-amino-3-hydroxy-5-methyl-4-isoxazolepropionic acid (AMPA) receptor trafficking to the postsynaptic membrane. This regulation significantly impacts synaptic plasticity and neuronal homeostasis [55, 56]. Another candidate was CACNA2D2, also known as alpha-2/delta subunit of the voltage-dependent calcium channel complex (α2δ2). This protein participates in the assembly and localization of a protein complex in the cell membrane, modulating calcium currents and the activation/inactivation kinetics of the channel. These processes regulate the entry of calcium ions into the cell after membrane polarization, which has important implications for neuronal function [57, 58]. Additional candidates included BRAF (B-Raf proto-oncogene, serine/threonine kinase, a protein within the RAF family of serine/threonine kinases that regulates the MAP kinase/ERK signaling pathway influencing cell division, differentiation and secretion [59]), MAPK9 (mitogen-activated protein kinase 9, a MAP kinase family member that participates in integrating biochemical signals and modulates proliferation, differentiation transcription regulation and development [60]), and GRK2 (G protein-coupled receptor kinase 2, a G protein-coupled receptor kinase that phosphorylates beta-adrenergic receptors and other substrates, including non-GPCR receptors, cytoskeletal proteins, mitochondrial components, and transcription factors [61]).

Brain homogenates from Tg2576 mice infused with young or old blood were used for western blotting analyses. While SYNGAP1, MAPK9 and GRK2 protein levels showed no significant differences between the two experimental conditions, the CACNA2D2 and BRAF protein levels were found to be increased in the brains of Tg2576 mice injected with old blood compared to Tg2576 mice injected with young blood (Figure 4G–4P). These data suggest that young and old blood infusion differentially affect synaptic plasticity and neuronal homeostasis, as well as the regulation and influx of cellular calcium ions in Tg2576 mice.

## DISCUSSION

In this study, we used young and old wild type animals as blood donors and Tg2576 mice at pre-pathological stages of brain amyloidosis as recipients to evaluate the effect of blood infusion on brain pathology. Our results showed an impairment in short- and long- term memory in Tg2576 transgenic mice injected with plasma from old wild type animals compared to those injected with plasma from young subjects (Figure 2). Regarding Aβ pathology, we observed significant differences in cortical regions where Tg2576 mice injected with the blood of old donors accumulated more Aβ compared with Tg2576 mice receiving young blood (Figure 3). These findings agree with previous studies showing that bloodborne factors present in old blood contribute to increased cognitive decline and impairments in synaptic plasticity [50, 62]. Along the same lines, it is well accepted that the administration of young blood has positive effects over multiple events, including aging and AD [63, 64]. Some studies have shown considerable decreases in Aβ deposits in transgenic animals treated with young blood [65, 66]. The above discussed reports and the data presented in this article indicate that old blood is attributed with factors that contribute to the “aging” of the recipient. Among the factors responsible for the aforementioned effects, several inflammatory molecules have been described. These include proinflammatory cytokines such as IL1β, IL6, IL27 and TNFα, as well as chemokines such as CCL11 and CCL27 [67]. In addition, molecules that facilitate lymphocyte trafficking to inflammatory sites in blood plasma have been identified [17, 68]. Another risk factor in aging is the accumulation of senescent uPAR+ cells, as these cells release proinflammatory cytokines such as PAI-1 and TGFβ, contributing to the reduction of immune cell proliferation and the transformation of niche cells into a proinflammatory phenotype [69, 70]. These processes and their effectors have been extensively studied [71]. All this information suggests that bloodborne factors can play an important role in AD pathology, with aged blood exerting a deleterious effect and potentially exacerbating AD pathology and reducing cognitive functions. It is noteworthy that, in the present study, the increase in Aβ burden among transgenic mice infused with old blood was exclusively detected employing the 4G8 antibody (Figure 3). Notably, ThS staining or biochemical evaluations by ELISA indicated no differences across the experimental groups (Supplementary Figures 1 and 3). While both ThS and the 4G8 antibody are employed to identify amyloid deposits, the dissimilarity in detection could potentially be attributed to the nature of the aggregates detected by each method. ThS binds to compact amyloid fibrils [72], while the 4G8 antibody specifically recognizes residues 17–24 within the Aβ sequence [73]. Consequently, 4G8 and ThS can effectively differentiate between various forms of aggregates [74, 75]. Along this line, blood infusion seems to alter the type of amyloid deposits in the brain. Specific for this study, the infusion of blood from old wild type donors to Tg2576 mice induced the accumulation of aggregates in the cortex. The mechanisms dictating the specific anatomical distribution of this phenotype will be characterized in future studies. Unfortunately, the biochemical analyses were not able to add more to these observations as they were conducted using homogenates prepared from the whole brain hemisphere.

Considering the cognitive and amyloid pathology alterations in these mice, we evaluated other components classically associated with brain amyloidosis. In the first place, we analyzed whether blood infusions altered glial markers. We observed no differences at this level (Supplementary Figures 4 and 5), suggesting that other mechanisms were involved in the modulation of pathological processes. Considering this, we analyzed whether the expression of APP could be altered in these mice, explaining the differences in amyloid deposition between the groups. In fact, significantly different increases in the presence of APP were observed in the brains of Tg2576 mice treated with the old blood (Figure 3), partially explaining the different degrees of amyloid pathology. The relevance of this finding to AD pathology is contentious, considering that human APP in this specific transgenic line is controlled by a non-physiological (prion protein) promoter [76].

To further study the mechanisms associated with pathological and memory cascades due to blood infusion, we analyzed the protein components altered in the mice included in this study. We conducted a proteomic analysis and found dysregulated components related to synaptogenesis and the endocannabinoid system (Figure 4). Based on the literature and the differentially expressed genes involved in AD, we validated the α2δ2 protein through western blotting in brain homogenates of transgenic mice injected with young and old blood. As suggested by the proteomic data, we found a significant increase in the levels of this protein in the brains of Tg2576 mice treated with old blood compared to Tg2576 mice treated with young blood. The α2δ2 protein is part of the voltage-gated calcium channels (VGCCs) protein complex, which belongs to the group of voltage-gated ion channels found in excitable cells, including neurons, allowing the permeability of calcium [77, 78]. In addition, the α2δ2 protein plays a pivotal role in the regulation of calcium-dependent signaling and neuronal excitability [75, 79]. Dysregulation of this protein has been linked to pathological conditions like hearing loss contributing to the facilitation of trans-synaptic alignment between presynaptic Ca^2+^ channels and postsynaptic AMPA receptors [80]. Interestingly, the α2δ2 subunit of VGCCs acts as a developmental switch that limits axon growth and regeneration. Removing or silencing *Cacna2d2*, which encodes the α2δ2 subunit, increases axon growth *in vitro* and enhances axon regeneration after spinal cord injury in adult mice [81]. These findings highlight the importance of understanding the dysregulation of α2δ2 in neurodegenerative diseases like AD, and the potential for therapeutic interventions targeting Aβ pathology and promoting axon regeneration. Additionally, our proteomic analysis revealed a significant decrease in the levels of BRAF in transgenic mice injected with young blood compared to those receiving old blood. The dysregulation of this protein has been linked with neurodegenerative processes, including tau hyperphosphorylation and neuronal dysfunction in AD [82, 83], as well as microglial proliferation and neuroinflammation [84, 85]. The upregulation of BRAF in animals treated with old blood compared to animals treated with young blood may reflect a proinflammatory environment characterized by increased cytokine activity, including IL1β, IL6 and TNFα, which have been implicated in synaptic dysfunction and neurodegeneration [86–88]. Previous studies have shown that young blood modulates BRAF signaling by reducing microglial activation and inflammatory pathways [89]. These findings reinforce the influence of systemic factors on intracellular signaling in AD and highlight BRAF as a potential target for therapeutic strategies aimed at mitigating neuroinflammation and synaptic impairment.

One limitation of our study involves the absence of a separate, blood-untreated control group. While our primary aim was to compare the effects of old versus young blood infusions on Tg2576 mice, the lack of a baseline reference group limits the interpretation of how either treatment diverges from the natural pathological progression in these animals. Future studies incorporating an untreated Tg2576 group or ideally, both transgenic and wild-type controls, will be essential to better contextualize the impact of bloodborne factors on disease progression. Despite this limitation, the direct comparison between old- and young- blood-treated groups provides valuable insights into the potential age-dependent effects of circulating factors in modulating AD-related pathology.

In summary, this study shows that blood from old and young mice carry elements able to modulate AD pathology and cognitive features. Interestingly, these changes appear to be specific to the cortical region and the type of deposits (compact vs. diffuse). Changes in memory due to blood infusion seem to be mediated by the α2δ2 protein as resolved through proteomic analyses and validated by western blotting. The identification of this protein mediating these events is a novel aspect of this study. We agree that future studies must confirm the proposed pathways. However, we believe that this study unveils different mechanisms as those previously described. The identification of neurodegeneration-relevant factors in blood is currently an active area of research with potential implications for the treatment of AD and other pathological conditions associated with aging. Further investigations are warranted to elucidate the specific factors responsible for these effects and to determine their potential translation to human treatments.

## MATERIALS AND METHODS

### Transgenic mice

The experiments described in this article used Tg2576 [52] and wild type littermates. Tg2576 mice express the human APP harboring the Swedish mutation. As a consequence, these mice start developing Aβ deposits in their brains at 8–9 months old and extensive presence of senile plaques and neuroinflammation at 17 months of age. Six to seven Tg2576 animals (random mixtures of males and females) were used per experimental group. Specifically, the group infused with blood from young wild-type donors included 3 males and 3 females (50% each), while the group infused with blood from old wild-type donors included 3 males and 4 females (42.9% males, 57.1% females). Although both sexes were represented, sex was not considered a biological variable in the statistical analyses due to the limited sample size.

### Intra-venous blood injections treatment

One hundred twenty-day old Tg2576 mice were immobilized using a restriction cell and injected with 150 μL of blood from wild-type mice in the tail vein using a ½ cc 27G ½ tuberculin syringe (BD Biosciences, Franklin Lakes, NJ, USA). Tg2576 mice received 30 blood doses separated by 7-day intervals. Blood treatments were performed using blood from young (50–75 days old) or old (443–532) wild-type mice. All treated animals were euthanized at 363–366 days old for subsequent analyses (Figure 1).

### Barnes maze test

To determine the spatial memory status of the experimental Tg2576 mice, the Barnes maze test was used [90]. The Barnes maze setup used in this study consisted of a circular table with 40 holes at their edges. One of these holes includes an escape box to exit the platform. The test is conducted by placing a single mouse in the center of the platform. Later, the mouse is stimulated with sound (loud clunk amplified by speakers) and the bright room’s light so they look to hide by going into the escape box. The escape box remains at the same position during the experiment and distinctive markings across the room are employed around the area as navigation cues. Mice were allowed to explore the arena for 3 minutes, 3 times a day, for 5 days. If they failed to find the escape box, they were gently directed to it by the researcher performing the study. Memory was assessed on the 5th and 12th days. The test on the 5th day reflects the short-term memory (STM), whereas that on the 12th day represents the long-term memory (LTM). Two parameters were measured in this test: time in latency (time to enter into the escaping chamber) and time in quadrant (percentage of time mice spent in the quadrant where the escape chamber is located).

### Histological analyses of brain slices

Tissue staining was performed as previously described [12, 19]. Briefly, brains were collected and one half of the brain (left) was frozen at −80°C for biochemical analyses, whereas the other half (right) was stored in 4% paraformaldehyde (PFA) and later paraffin embedded and cut for histological studies. Serial, 10 μm-thick sections from groups of Tg2576 animals transfused with old and young blood were processed for histological analyses. For immunohistochemistry, serial sections were deparaffinized and hydrated in decreasing ethanol gradients. Endogenous peroxidase activity was blocked with 3% H_2_O_2_/10% methanol in PBS for 20 min. In the case of needing antigenic unmasking of the epitope, 85% v/v formic acid was used for 3 minutes. The primary antibodies used were 4G8 Mouse IgG2b (Biolegend, San Diego, CA, USA) (1:1000), and anti-GFAP Rabbit/IgG (Abcam, Fremont, CA, USA) (1:1000). For 4G8 and anti-GFAP primary antibodies, the incubation time was overnight. All antibody incubations were performed at room temperature, and the subsequent washings were performed using PBS to remove antibody excess. A 2-hours incubation was performed at room temperature with corresponding secondary antibodies bound horseradish peroxide (HRP): Goat anti-mouse (Jackson ImmunoResearch West Grove, PA, USA) (1:1000), or Goat anti-rabbit (Jackson ImmunoResearch, West Grove, PA, USA) (1:1000). The peroxidase reaction was visualized using the DAB Peroxidase Substrate Kit (Vector Labs, Newark, CA, USA) following the manufacturer’s instructions. Finally, the tissue slices were subjected to dehydration in increasing ethanol gradients (70%-100%), xylene clearance, and coversliped with ENTELLAN mounting solution (Sigma-Aldrich, Saint Louis, MO, USA). Additionally, brain sections were incubated with Thioflavin-S (ThS) solution (0.025% in 50% ethanol) for 10 min and dehydrated and mounted with ENTELLAN or Fluoromount (Electron Microscopy Sciences, Hatfield, PA, USA). All images were visualized and captured using Eclipse E200 series 624721 binocular microscopes (Nikon Minato-ku, Tokyo, Japan) in bright field and 488–555 nm filters. Between 4 and 5 tissue slices per animal/staining, were taken every 10 slices and used for image analyses and quantifications. Burden was defined as the labeled area of the brain per total area analyzed, and the results were expressed as percentage. The region analyzed corresponded to the entire cortical and hippocampal areas of the sections studied. Histological staining and image analyses were conducted using the Fiji ImageJ Win-64 Software.

### Quantification of Aβ levels by ELISA

Brain tissue extracts from experimental groups were processed following a standardized protein extraction protocol [75]. Briefly, brain homogenates (10% w/v) were prepared in PBS and centrifuged at 32,600 rpm for 1 hour at 4°C using a Sorvall WX100 ultracentrifuge (Thermo Fisher, Norristown, PA, USA) equipped with a Fiberlite fixed-angle rotor F50L-24 × 1.5 (Thermo Fisher, Norristown, PA, USA). The supernatants were saved and designated as the PBS-soluble fractions. The remaining pellets were resuspended in 2% sodium dodecyl sulphate (SDS) and homogenized by pipetting followed by sonication in a water bath until complete solubilization. After a second round of centrifugation under the same conditions, the SDS-soluble fractions were collected. The remaining pellets were then treated with 70% formic acid (Fisher Scientific, Waltham, MA, USA) and subjected to sonication (water bath) until full dispersion. The FA-solubilized samples underwent centrifugation for 30 minutes, and the resulting supernatants were collected as FA fractions. To neutralize acidity, FA fractions were diluted 1:20 in 1 M Tris buffer pH 11 (Sigma Aldrich, Saint Louis, MO, USA). Aβ_42_ and Aβ_40_ peptides present in these samples were measured by using ELISA Aβ_42_ and Aβ_40_ kits (Invitrogen, Carlsbad, CA, USA). ELISA was performed following the manufacturer’s instructions.

### Western blot analyses of brain homogenates

The western blot procedure was performed as described elsewhere [91]. Briefly, brain homogenate samples were lysed with PBS supplemented with a protease inhibitor cocktail (Sigma-Aldrich, Saint Louis, MO, USA) plus a phosphatase inhibitor cocktail (Sigma-Aldrich, Saint Louis, MO, USA). Protein concentration was measured using the Qubit dsDNA BR assay kit (Thermo Fisher, Norristown, PA, USA) following the manufacturer’s instructions. Twenty μg/μL of samples were loaded onto 10% polyacrylamide gels under denaturing conditions (sodium dodecyl sulfate-polyacrylamide gel electrophoresis (SDS-PAGE)) and transferred to nitrocellulose membranes. The membranes were incubated with the following primary antibodies overnight at 4°C in agitation: 6E10 mouse/IgG1 (Biolegend, San Diego, CA, USA) (1/500), α2δ2 (*Cacna2d2*) rabbit/IgG (Abcam, Fremont, CA, USA) (1:1000), SynGAP1 rabbit/IgG (Abcam, Fremont, CA, USA), BRAF rabbit/IgG (Proteintech, Rosemont, IL, USA), JNK2 (MAPK9) mouse/IgG (Origene, Rockville, MD, USA), GRK2 mouse/IgG1 (Invitrogen, Carlsbad, CA, USA), and β-Actin mouse/IgG2b (Cellsignal, Danvers, MA, USA). Then, the following peroxidase-conjugated secondary antibodies were used: Goat anti-mouse (Jackson ImmunoResearch West Grove, PA, USA) (1:5000), Goat anti-rabbit (Jackson ImmunoResearch West Grove, PA, USA) (1:5000). Incubations with secondary antibodies were conducted for 1 h at room temperature. Immunoreactivity was visualized using the ECL Plus^™^ detection system (GE Healthcare, Chicago, IL, USA). Densitometric quantification of the bands was performed using the ImageJ Software.

### Proteomic analysis

Brain homogenates from blood-treated Tg2576 mice were sent to MELISA Institute (San Pedro de la Paz, Bio Bio, Chile) for analysis. The detailed workflow was performed according to the institute’s own parameters. In essence, the workflow began as follows. Protein extraction and trypsin treatment: each sample was treated with a protease/phosphatase inhibitor (Thermo Fisher, Norristown, PA, USA) at a 1X concentration. After lyophilization, samples were resuspended in 8 M urea and 25 mM ammonium bicarbonate at pH 8, followed by ultrasonic homogenization for 1 minute at 50% amplitude with 10-second pulses in a cold bath. Debris were removed through centrifugation at 21,000 × *g* for 10 minutes at 4°C. Protein quantification was carried out using Qubit Protein Assay reagent (Thermo Fisher, Norristown, PA, USA). Preparation for mass spectrometry (MS): proteins underwent chloroform/methanol extraction, as previously described [92]. Following equilibration, centrifugation, and removal of the supernatant, the protein pellet underwent thrice washing with cold 80% acetone and was subsequently dried. The pellet was then resuspended in 30 μL of a buffer made of 8 M urea, 2% SDS, 2% deoxycholate in 25 mM ammonium bicarbonate pH 8. Reduction of proteins’ disulfide bonds was carried out by incubating the samples for 30 min with dithiothreitol (DTT), followed by alkylation (by incubating the sample for 30 min with 25 mM iodoacetamide). Then, the samples were diluted 8 times with 25 mM ammonium bicarbonate pH 8, and digested with sequence quality trypsin (Promega, Madison, WI, USA) in a 1:50 ratio, overnight at 37°C. Clean Up Sep-Pak C18 Spin Columns (Sigma-Aldrich, Saint Louis, MO, USA) were employed for cleanup, and the resulting clean peptides were dried. Database searching tandem mass spectra: This was extracted by Tims Control version 2.0 (Burker Daltonic Billerica, MA, USA). Charge state deconvolution and deisotoping were not performed. All MS/MS samples were analyzed using PEAKS Studio (Bioinformatics Solutions, Waterloo, ON Canada; version 10.5 (2019-11-20)). PEAKS Studio was set up to search the (UniProt_SwissProt) database (21040 entries) assuming an efficient trypsin digestion. PEAKS Studio was searched with a fragment ion mass tolerance of 0,050 Da and a parent ion tolerance of 50 PPM. Carbamidomethyl of cysteine was specified in PEAKS Studio as a fixed modification. Deamidated of asparagine and glutamine, oxidation of methionine, acetyl of the n-terminus and carbamyl of lysine and the N-terminus were specified in PEAKS Studio as variable modifications.

### LFQ and differential expression analysis

Individual identification reports from PEAKS were concatenated, and missing values (NA) results were imputed by MICE [93]. To determine which proteins were differentially and significantly expressed in the treatment contrast we applied a Wald test to data with a Benjamini-Hochberg correction using Deseq2 [94]. Any protein associated with *p*-adjust <0.05 was considered significant. Graphic representations related to quantification results were created using statistical environment R v.3.6.0 [95] with EnhancedVolcano [96], Complex Heatmap v.2.0.0 [97], GOplot [98] and base packages of R.

### Bioinformatic analysis

The proteomic dataset including UniProt identifiers and logFC values of identified proteins in mass spectrometry was submitted to ingenuity pathway analysis (IPA). Networks, functional analyses and pathways were obtained through the use of IPA (QIAGEN Inc., https://digitalinsights.qiagen.com/IPA) [99]. Core analysis was performed with the following settings: (i) indirect and direct relationships between molecules, (ii) based on experimentally observed data, and (iii) all data sources were admitted from the Ingenuity Knowledge Base.

### Statistical analysis

To evaluate differences between groups, normality was first assessed using the Shapiro-Wilk test (α = 0.05), and homoscedasticity was evaluated using Levene’s test for normally distributed data or the Fligner-Killeen test for non-normal data (α = 0.05). Outliers were detected using the interquartile range (IQR) method and were either removed or transformed. Group comparisons were performed with two-tailed *t*-tests for normally distributed data with homoscedasticity. Non-normally distributed data were analyzed with non-parametric tests, such as the Mann-Whitney *U*-test. For repeated measures data, normality was assessed per block using the Shapiro-Wilk test, and homogeneity of variances was evaluated with Levene’s test. If data met normality assumptions and variances were homogeneous, a repeated-measures ANOVA was performed to evaluate the effect of time (blocks) and group differences. In cases where data deviated from normality, a non-parametric alternative, such as the Friedman test, was considered. If significant differences were detected, post-hoc comparisons were conducted using the Tukey’s test for ANOVA or the Dunn-Bonferroni for the Friedman test. All these analyses were conducted using α = 0.05. The statistical analyses and graphical representations were performed using R (v 4.4.2) with the car (v 3.1-3), nlme (v 3.1-162), and rstatix (v 0.7.2) packages, as well as the GraphPad Prism software (v 8.0.1, GraphPad Software Inc., Boston, MA, USA).

## Supplementary Materials

Supplementary Figures

Supplementary Data 1
