# Supplementary material for: Infusion of blood from young and old mice modulates amyloid pathology
Source: Aging (Albany NY). 2025 Sep 12;17(11):2664–87. doi: 10.18632/aging.206319 (PMC12705187; doi:10.18632/aging.206319)
Supplement: Supplementary Figures [file aging-17-11-206319-s001.pdf]

SUPPLEMENTARY FIGURES

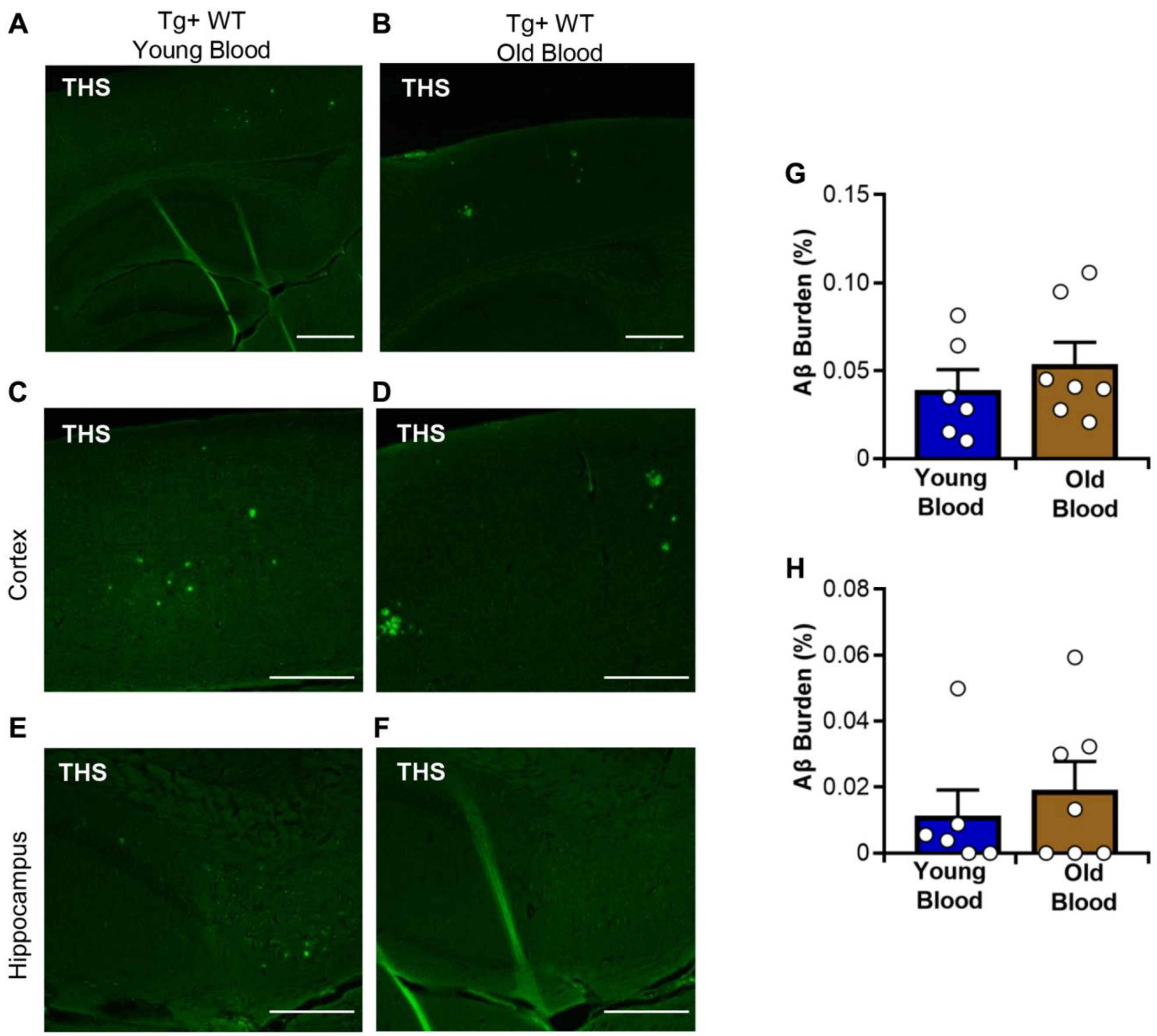

Supplementary Figure 1. Deposition of ThS reactive amyloid plaques in the brains of Tg2576 treated with blood from old and young wild type mice. Representative images of the accumulation of ThS positive amyloid deposits in brains of blood treated Tg2576 mice. (**A**, **B**) The brain regions used for analyses included cortex (**C**, **D**) and hippocampus (**E**, **F**). Quantitative analyses of ThS burden in cerebral cortical (**G**) and hippocampal (**H**) sections. Scale bars: 500 µm. *N* = 5–7/group, (random mix of males and females; young donor group: 3M/3F; old donor group: 3M/4F). Data values are expressed as mean ± SEM. Data in (**G**) were analyzed using Student's *t*-test, and data in (**G**) were analyzed using the Mann-Whitney *U*-test.

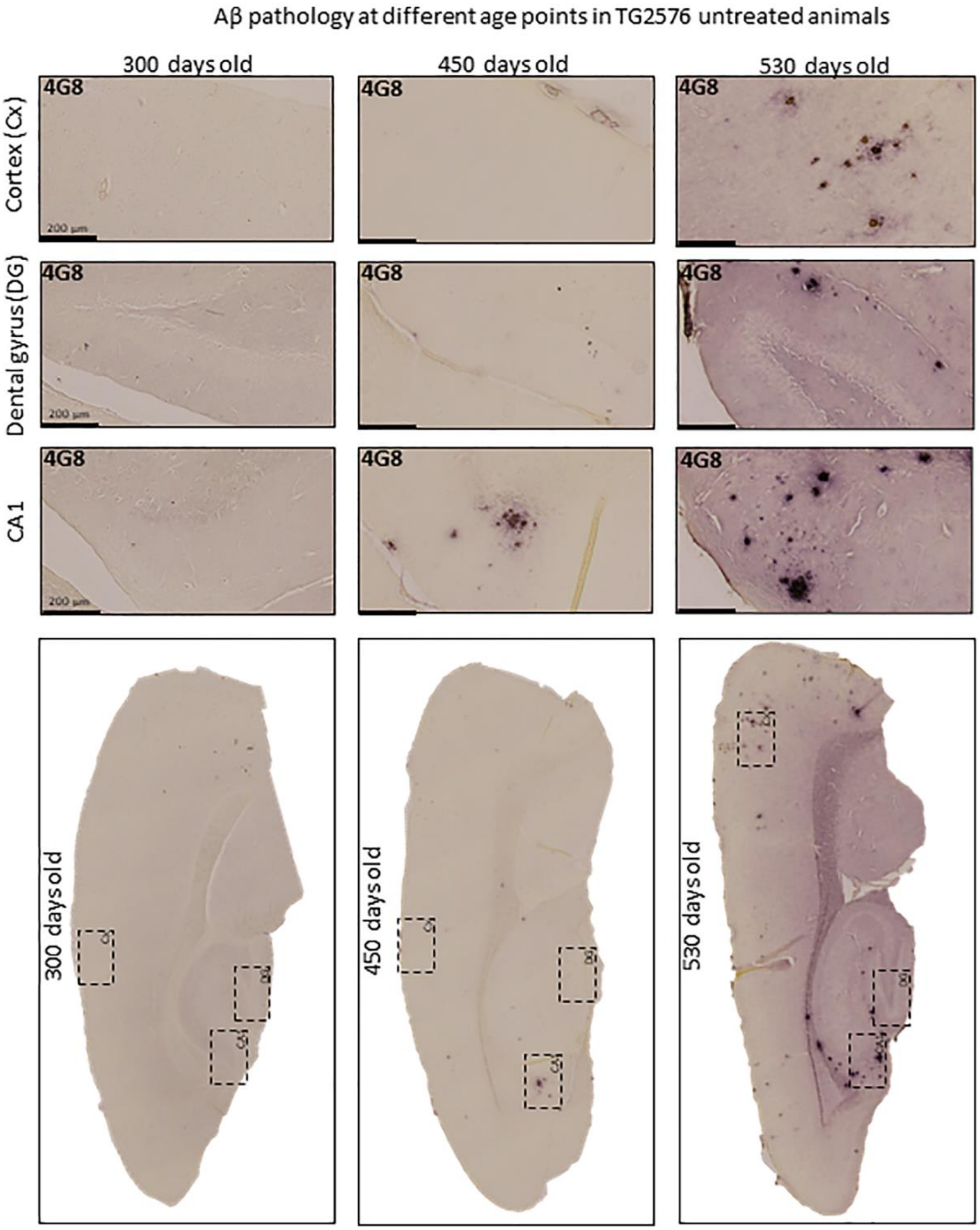

Supplementary Figure 2. Time-course propagation of Aβ pathology in Tg2576 mice. This figure depicts the time-dependent increase of amyloid pathology over time in the brain of Tg2576 mice. Tissues were collected in 300-, 450-, and 530-day-old Tg2576 mice. Three different brain regions are enlarged for better visualization: cortex, dentate gyrus, and CA1. Scale bar: 200 µm.

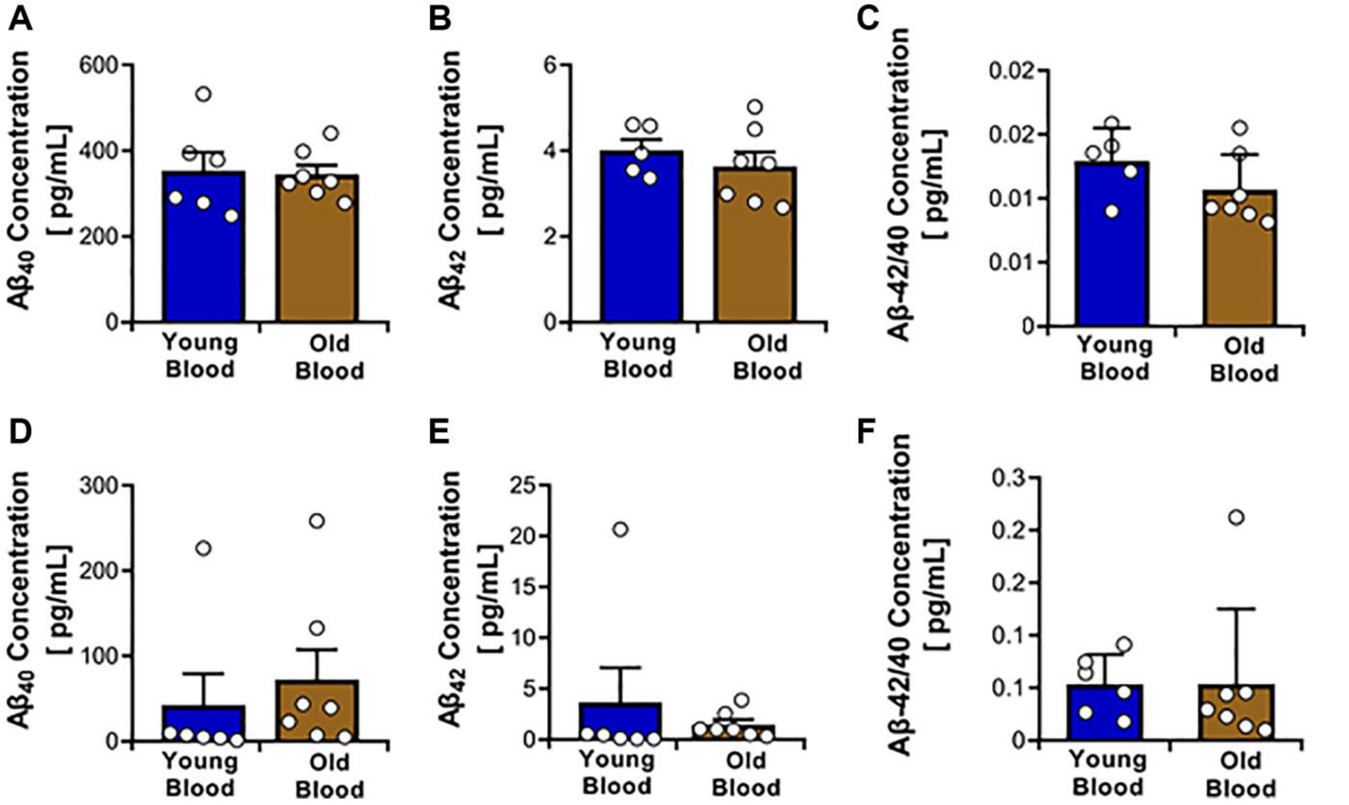

Supplementary Figure 3. Quantification of Aβ_{40} and Aβ_{42} levels in PBS and formic acid (FA) fractions from brain homogenates of Tg2576 mice treated with young or old blood. Aβ_{40} and Aβ_{42} peptide concentrations were measured using ELISA in different brain extract fractions. (**A**) Aβ_{40} concentration in the PBS fraction. (**B**) Aβ_{42} concentration in the PBS fraction. (**C**) Aβ_{42}/Aβ_{40} ratio in the PBS fraction. (**D**) Aβ_{40} concentration in the FA fraction. (**E**) Aβ_{42} concentration in the FA fraction. (**F**) Aβ_{42}/Aβ_{40} ratio in the FA fraction. *N* = 6–7/group, (random mix of males and females; young donor group: 2–3M/2–3F; old donor group: 2–3M/2–4F). Data values are expressed as mean ± SEM. Statistical analysis was performed as follows: (**A**, **D**, **E**, **F**), Mann-Whitney *U*-test; (**B**, **C**), Student's *t*-test.

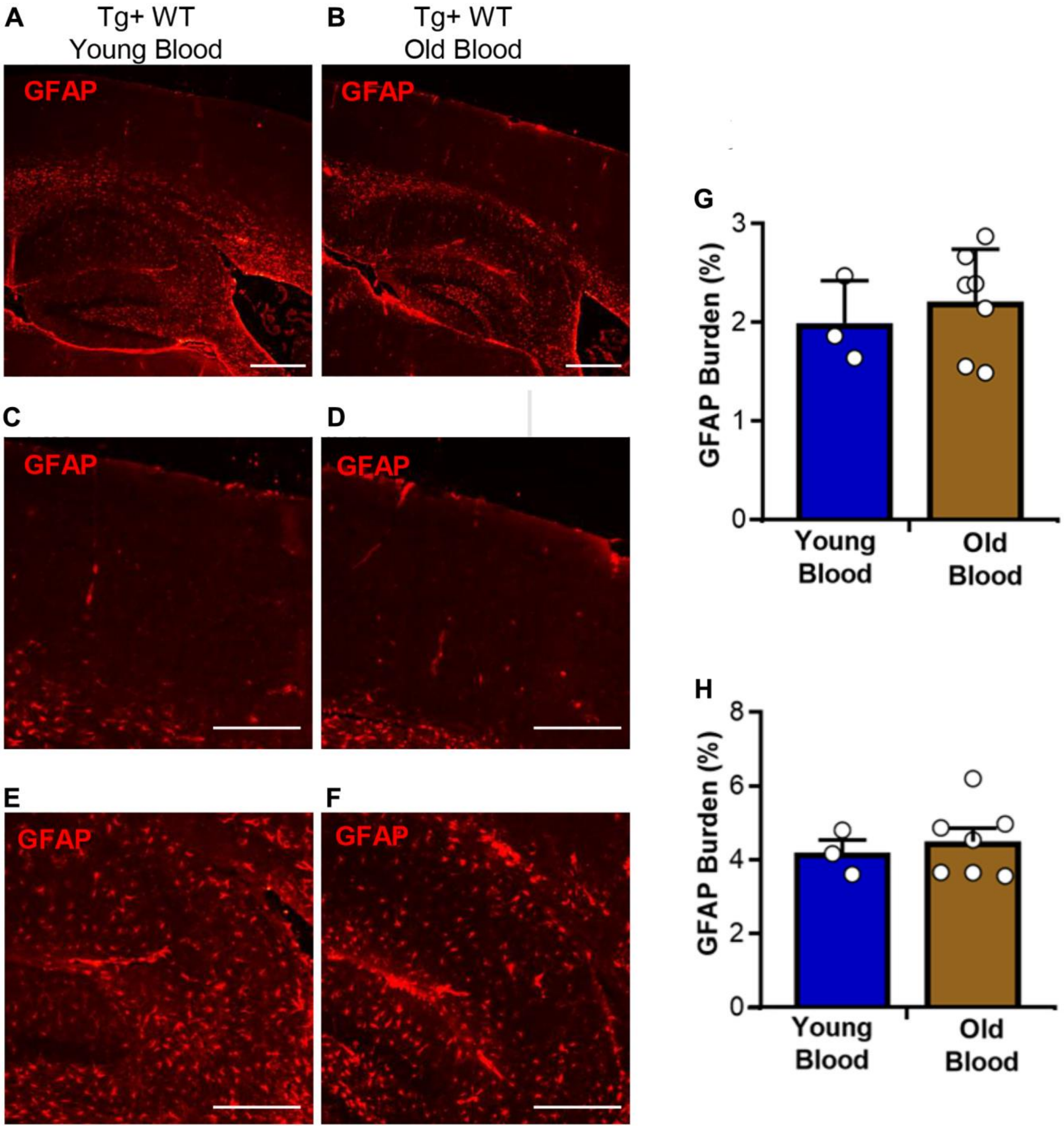

Supplementary Figure 4. Histopathological analysis of an astrocyte marker in the brain of Tg2576 mice infused with blood from old and young wild type mice. Representative images displaying the presence of the astrocyte cell marker GFAP in the brain of Tg2576 mice. (**A**, **B**) The brain regions used for analysis included cortex (**C**, **D**) and hippocampus (**E**, **F**). Quantitative analyses of GFAP burden in cerebral cortical (**G**) and hippocampal (**H**) sections are shown. Scale bars: 500 µm. *N* = 5–7/group, (random mix of males and females; young donor group: 3M/3F; old donor group: 3M/4F). Data values are expressed as mean ± SEM. Data in (**G**, **H**) were analyzed using Student's *t*-test.

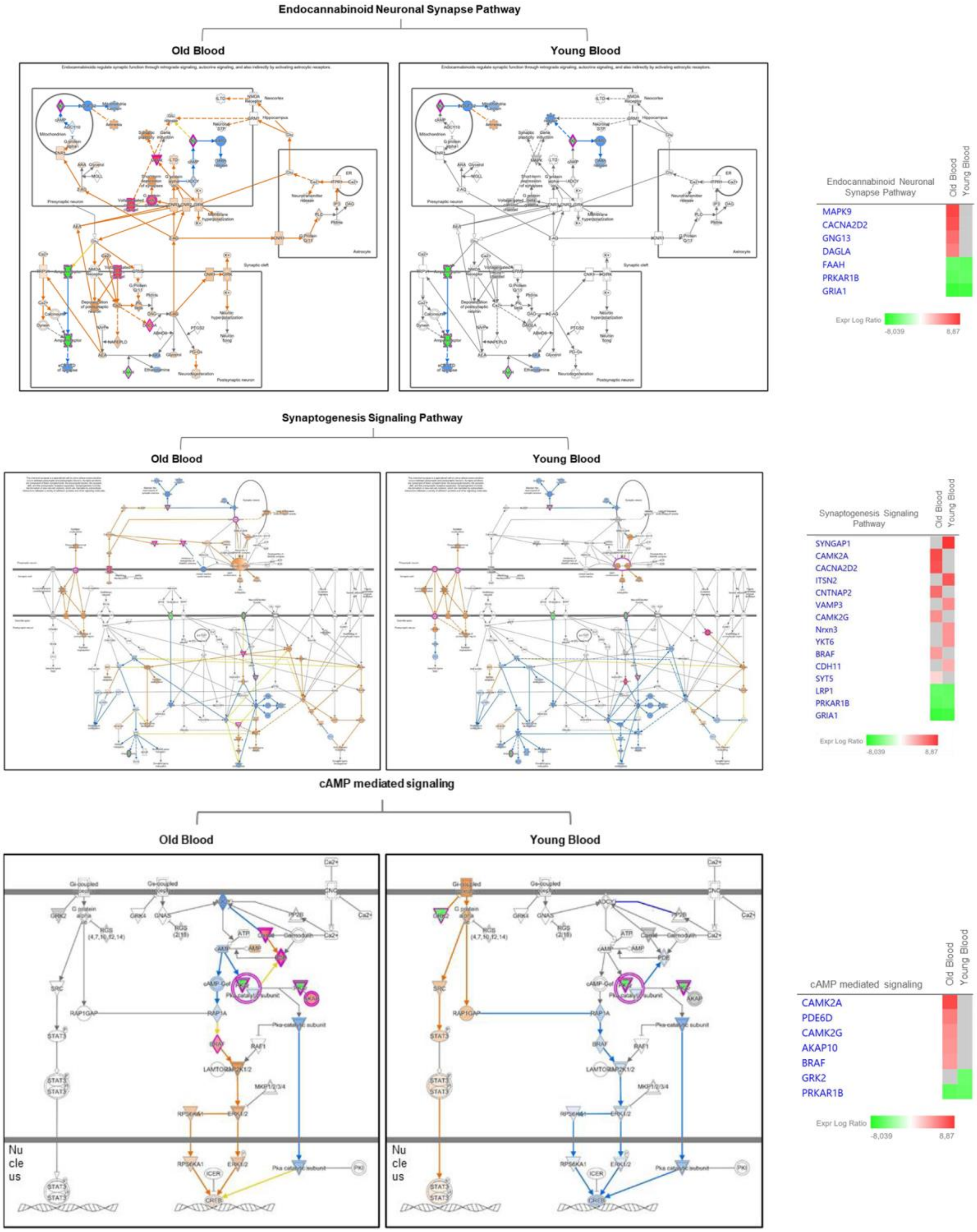

Supplementary Figure 5. Proteomic pathways analysis of Tg2576 mice infused with blood from old and young wild type mice. Pathway analysis of differentially expressed proteins in cAMP-mediated signaling, synaptogenesis signaling, and endocannabinoid neuronal synapse pathways in the Old Blood and Young Blood groups. Pathway activation is represented by orange (activation) and blue (inhibition) connections. Differentially expressed proteins within each pathway, with color-coded log expression ratios are displayed. Gene names are used. *N* = 3/group, (random mix of males and females; young donor group: 1M/2F; old donor group: 1M/2F).
